# Effect of prevalence of alcohol consumption and tobacco use in Mexican municipalities on early childhood development

**DOI:** 10.1590/0102-311XEN112422

**Published:** 2023-12-11

**Authors:** Francisco-Javier Prado-Galbarro, Carlos Sanchez-Piedra, Juan-Manuel Martínez-Núñez

**Affiliations:** 1 Departamento de Sistemas Biológicos, Universidad Autónoma Metropolitana-Unidad Xochimilco, Ciudad de México, México.; 2 Dirección de Investigación, Hospital Infantil de México Federico Gómez, Ciudad de México, México.; 3 Agencia de Evaluación de Tecnologías Sanitarias, Instituto de Salud Carlos III, Madrid, España.

**Keywords:** Child Development, Child Welfare, Alcohol Consumption, Tobacco Use, Neighborhood, Desarrollo Infantil, Protección a la Infancia, Consumo de Bebidas Alcohólicas, Uso de Tabaco, Vecindarios, Desenvolvimento Infantil, Proteção da Criança, Consumo de Bebidas Alcoólicas, Uso de Tabaco, Vizinhança

## Abstract

One of the most critical time periods in childhood is from birth to five years of age. Children exposed to alcohol and/or tobacco via family members and neighborhood are at risk for childhood developmental delays. This study evaluated the association of early childhood development with the prevalence of alcohol consumption and tobacco use in Mexican municipalities. This is a cross-sectional study. Early childhood development information from 2,345 children aged from 36 to 59 months was obtained from the 2015 *Mexican National Survey of Boys, Girls, and Women* (ENIM). Data on alcohol consumption and tobacco use come from the 2016 *Mexican National Survey on Drugs, Alcohol, and Tobacco Consumption* (ENCODAT). Multilevel logistic models were fitted to evaluate the association of the prevalence of alcohol consumption and tobacco use with the inadequacy of early childhood development. Children living in municipalities with high prevalence of alcohol consumption (OR = 13.410; 95%CI: 2.986; 60.240) and tobacco use (OR = 15.080; 95%CI: 2.040; 111.400) were less likely to be developmentally on track regarding early childhood development after adjustment for individual variables related to the child’s development and other environmental variables at municipal level. Childhood exposure to alcohol and tobacco in the neighborhood may directly contribute to inadequate early childhood development. These findings suggest that there is an urgent need to develop effective interventions aimed at reducing alcohol consumption and tobacco use in municipalities to ensure adequate early childhood development.

## Resumen

El grupo de edad que se extiende desde el nacimiento hasta los 5 años es uno de los periodos más críticos en la infancia. Niños expuestos al alcohol y/o al tabaco a través de la familia y vecinos corren el riesgo de sufrir un retraso en el desarrollo infantil. Este estudio evaluó la asociación del desarrollo durante la primera infancia con la prevalencia del consumo de alcohol y tabaco en municipios mexicanos. Se trata de un estudio transversal. Las informaciones sobre el desarrollo durante la primera infancia de 2.345 niños de 36 a 59 meses se obtuvieron a través de la *Encuesta Nacional de los Niños, Niñas y Mujeres en México* (ENIM) de 2015. Los datos sobre el consumo de alcohol y tabaco son de la *Encuesta Nacional de Consumo de Drogas, Alcohol y Tabaco en México* (ENCODAT) de 2016. Se ajustaron los modelos logísticos multiniveles para evaluar la asociación de la prevalencia de consumo de alcohol y tabaquismo con desarrollo durante la primera infancia inadecuado. Los niños que viven en municipios que tienen una alta prevalencia de consumo de alcohol (OR = 13,410; IC95%: 2,986; 60,240) y tabaquismo (OR = 15,080; IC95%: 2,040; 111,400) se asociaron con la probabilidad más alta de tener un desarrollo durante la primera infancia inadecuado tras el ajuste de las variables individuales relacionadas al desarrollo del niño y a otras variables ambientales en nivel municipal. La exposición infantil al alcohol y al tabaco en la vecindad puede contribuir directamente a un desarrollo durante la primera infancia inadecuado. Estos hallazgos indican una necesidad urgente de desarrollar intervenciones eficaces destinadas a reducir el consumo de alcohol y tabaquismo en los municipios para asegurar un desarrollo durante la primera infancia adecuado.

## Resumo

A faixa etária que se estende do nascimento aos 5 anos de idade é um dos períodos mais críticos na infância. Crianças expostas ao álcool e/ou tabaco por meio de familiares e vizinhos estão em risco de atraso no desenvolvimento infantil. Este estudo avaliou a associação do desenvolvimento durante a primeira infância com a prevalência do consumo de álcool e tabaco em municípios mexicanos. Trata-se de um estudo transversal. As informações sobre o desenvolvimento durante a primeira infância de 2.345 crianças de 36 a 59 meses foram obtidas pela *Pesquisa Nacional Sobre Crianças e Mulheres no México* (ENIM) de 2015. Os dados sobre consumo de álcool e tabaco são da *Pesquisa Nacional sobre Consumo de Drogas, Álcool e Tabaco no México* (ENCODAT) de 2016. Modelos logísticos multiníveis foram ajustados para avaliar a associação da prevalência de consumo de álcool e tabagismo com desenvolvimento durante a primeira infância inadequado. Crianças que vivem em municípios com alta prevalência de consumo de álcool (OR = 13,410; IC95%: 2,986; 60,240) e tabagismo (OR = 15,080; IC95%: 2,040; 111,400) foram associadas à maior probabilidade de ter um desenvolvimento durante a primeira infância inadequado após ajuste às variáveis individuais relacionadas ao desenvolvimento da criança e a outras variáveis ambientais em nível municipal. A exposição infantil ao álcool e tabaco na vizinhança pode contribuir diretamente para o desenvolvimento durante a primeira infância inadequado. Estas descobertas demonstram uma necessidade urgente de desenvolver intervenções eficazes destinadas a reduzir o consumo de álcool e tabagismo nos municípios para garantir um desenvolvimento durante a primeira infância adequado.

## Introduction

Child development is a public health priority worldwide, included as one of the United Nations Sustainable Development Goals ^1^. The period from birth to five years of age is considered the most important developmental phase of humans and it is a key stage to improve individual’s life trajectories ^2^ ^,^ ^3^ ^,^ ^4^. An adequate early childhood development strongly influences well-being throughout life ^3^. It is estimated that more than 200 million children under five years of age from low- and middle-income countries are at risk of not achieving their full developmental potential due to several lacks and barriers related to the environments responsible for fostering child development, such as family, school, and civil society in a broader context ^5^.

Generally, research regarding child development has focused on the proximal determinants, mainly describing the influence of individual, maternal, familiar, and school predictors on child’s growth and development. There is evidence regarding the role of maternal nutrition, breastfeeding, parenting skills, parent’s education, cultural practices, and intra-family relations on child development ^3^ ^,^ ^6^. There is also ample evidence of associations between socioeconomic conditions of the family and child development ^7^ ^,^ ^8^ ^,^ ^9^. For instance, maternal educational attainment is strongly associated with better child development: children of mothers with low educational level may have a slower physical and emotional development when compared with children of mothers with complete high-school or higher education ^10^ ^,^ ^11^. It has been proposed that mothers and families need supportive environments to be able to provide opportunities that allow their children to fulfill their potentialities. The neighborhood environment has been identified as key for supporting families to overcome adversities that could constrain child development ^3^ ^,^ ^12^ ^,^ ^13^. Neighborhood residential instability in childhood can contribute to the development of major depressive disorder and antisocial personality disorder ^14^.

As in other countries, alcohol consumption and tobacco use are two of the major public health problems in Mexico, especially in reproductive age people. The consumption of alcohol and tobacco use presents repercussions on the consumer’s family and social environment, including the development of children in early childhood ^15^. The family is considered the central axis in the early childhood development process since it is the closest and most immediate environment to the child. The influence family exerts throughout childhood can last a lifetime ^16^.

Several studies have analyzed the effect of alcohol consumption and tobacco use during pregnancy on children’s behavioral disorders ^17^ ^,^ ^18^. However, these studies have not considered exposure to alcohol and tobacco in neighborhoods as an important risk factor for children’s development. In this study, we explored the association between early childhood development and the prevalence of alcohol and tobacco use in Mexican municipalities, adjusting individual and family-level determinants. The main hypothesis was that children living in municipalities with higher prevalence of alcohol and tobacco use, along with those with higher levels of crime and marginalization, could present more developmental issues.

## Methods

### Study design, participants, and setting

This is a retrospective study with information extracted from the 2016 *Mexican National Survey on Drugs, Alcohol, and Tobacco Consumption* (ENCODAT - Encuesta Nacional de Consumo de Drogas) and the 2015 *Mexican National Survey of Boys, Girls, and Women* (ENIM - Encuesta Nacional de los Niños, Niñas y Mujeres). These surveys used probabilistic multistage stratified cluster sampling design and are representative at the national and regional levels and for urban/rural areas. Furthermore, ENCODAT is also representative at the state level.

ENIM 2015 was part of the fifth round of the *Multiple Indicator Cluster Survey* (MICS 5), and oversampled children under age five and rural areas to estimate indicators for children and women ^19^. Four questionnaires were applied to get information on: (a) the household, (b) women aged 15 to 49, (c) children and adolescents aged 5 to 17 years (answered by their mothers), and (d) children under age five (answered by their mothers).

The municipality of residence of children who participated in the ENIM 2015 was linked to its prevalence of alcohol consumption and tobacco use, along with other secondary sources, in the same area through unique municipality codes. A total of 2,345 children belonging to 200 municipalities were selected.

### Outcome variable: early childhood development

The primary source of early childhood development data was the Early Childhood Development Index (ECDI), which is a population-based measure developed and validated by the MICS program from the United Nation’s Children Fund (UNICEF) during 2006-2009. The ECDI was conducted in around 80 low- and middle-income countries, being considered a useful tool for identifying inadequate development in children aged 36-59 months.

The ECDI represents the percentage of children aged 36-59 months who were developmentally on track (adequate early childhood development) in at least three out of the following four domains: literacy-numeracy, physical development, social-emotional development, and learning. The ECDI is composed of 10 items, each with yes/no responses, covering all domains ^20^. Based on ECDI criteria, literacy-numeracy development is considered on track if children can do at least two of the following: identify 10 or more letters of the alphabet; read four or more common simple words; and/or name and recognize all the numbers from 1 to 10. The social-emotional development is considered on track if children can do at least two of the following: get along well with other children; do not get distracted easily, and/or do not hit, bite, or kick other children. The physical development is considered on track if at least one of the following is true about the children: can pick up small objects (like a stick or rock from the ground) with two fingers and/or does not often feel sick to play. Learning development is considered to be on track if children can do at least one of the following: can follow simple instructions on how to do something correctly and/or when given something to do, is able to do it independently. Therefore, each domain was considered as a binary outcome, and children aged 36-59 months were considered to be developmentally on track if they met the age-expected development targets in at least three out of the four domains.

### Exposure variables: alcohol consumption and tobacco use

The prevalence of alcohol consumption and tobacco use by municipality was estimated by implementing small area estimation with ENCODAT data. Auxiliary information for small areas was aggregated to increase survey sampling precision. Area-level models described by Fay-Herriot were used ^21^, which is a widely used technique to obtain reliable estimates for small areas. Then, estimated averages from the outcomes of interest were combined with secondary data for each municipality, such as crime rate ^22^, social backwardness index ^23^, and population density ^24^.

### Covariates

(i) Child-level covariates: the analysis incorporated the following information on children: sex, age in months, presence of any functional difficulty (seeing, hearing, walking, fine motor coordination, understanding, being understood, learning things, playing, and, where applicable, of controlling behavior), maternal age, maternal education, indigenous origin, and households’ wealth quintiles.

(ii) Municipality-level covariates: population density ^24^ and number of reported homicides ^25^, obtained from the Mexican Institute of Geography and Statistics (INEGI). It uses data from death certificates, which tends to be more precise than Mexican police records.

### Statistical analysis

Individual and municipal characteristics by early childhood development status were analyzed using percentages and 95% confidence intervals (95%CI) for categorical variables, along with means and standard deviations (SD) for continuous variables. Moreover, bivariate analyses were performed to identify the association between individual- and municipal-level variables and early childhood development. Then, to test the association between the proportion of alcohol consumption and early childhood development, as well as the proportion of daily tobacco smokers and early childhood development, multilevel multivariable logistic regression models were used with data from individuals nested within municipalities. A series of models were fitted: two models including each exposure separately, and a final model including all exposures together. Then, these models were adjusted for each child- or municipal-level variable with a p-value ≤ 0.20 in the bivariate analysis. The following multivariate regression model was used:

$$y_{ij}∼Binomial\left(1,\pi_{ij}\right)$$

$$og\left(\frac{\pi_{ij}}{1-\pi_{ij}}\right)=\beta_{0}+\sum_{k=1}^{n}\beta_{k}X_{ki}+\sum_{l=1}^{m}\beta_{l}Z_{lj}+u_{j}$$

Where *π*_*ij*_ is the probability of inadequate early childhood development for child *i* in municipality *j*, *X*_*i*_ is the set of explanatory variables at the child-level (sex, age in months, functional difficulties, maternal age, maternal education, household wealth quintiles, indigenous origin, and domestic violence), *Z*_*j*_ is the set of explanatory variables defined for the municipalities (proportion of alcohol consumption/proportion of daily tobacco smokers, number of homicides and population density), and *u*_*j*_ are the residuals of level 2, for which it is assumed that they are independent and follow a normal distribution with mean 0 and a variance of $\sigma_{u}^{2}$ .

The median odds ratio (OR) were computed to quantify the effect of area-level variance, which can be interpreted as the increased risk (in median) of having inadequate early childhood development if the child moves from the municipality at lowest risk to the municipality at highest risk ^26^. The median OR is equal to 1 when there is no variation between municipalities. However, if the median OR is greater than 1, then between-municipality variation is significant.

All analyses were performed in Stata, version 16.0 (https://www.stata.com). Estimates of descriptive indicators account for the complex survey design and survey weights. In statistical models, weighted multilevel logistic models were estimated by child-level weights at level 1 (pweight). Statistical tests were two-tailed and considered significant below a 0.05 alpha.

### Ethics approval and informed consent

During the data collection of the ENIM and ENCODAT, the participants consented to voluntary and anonymously participate by signing a consent letter. Both studies were conducted in accordance with the *Declaration of Helsinki*. All analyses in this study were performed with de-identified secondary data. Since it was a secondary data analysis, this study did not require the approval of a Research Ethics Committee.

## Results

A total of 2,345 children aged 36-59 months were analyzed. The study sample has a mean age of 48.71 months (SD: 7.08), 56.41% of the sample was female, 1.3% had functioning difficulties, and 39.6% lived in a poor or very poor household. At baseline, 41.61% of the mothers had complete middle school with a mean age of 29.63 years (SD: 6.59), and 23.27% were indigenous (Table 1).

**Table 1:** Individual characteristics of children with inadequate and adequate early childhood development.

Characteristics	Early childhood development [% (95%CI)]		p-value	Total [% (95%CI)]
	Inadequate	Adequate		
Total [n (%)]	484 (17.44)	1,861 (82.56)		2,345 (100.00)
Functional difficulties				
No	17.14 (14.07; 20.70)	82.86 (79.30; 85.93)	< 0.001	98.70 (98.06; 99.13)
Yes	40.22 (26.07; 56.20)	59.78 (43.80; 73.93)		1.30 (0.87; 1.94)
Maternal education				
Elementary or less	21.72 (16.9; 27.46)	78.28 (72.54; 83.10)	0.012	16.44 (13.49; 19.89)
Middle school	21.23 (17.3; 25.78)	78.71 (73.12; 83.40)		41.61 (35.29; 48.22)
High school	16.49 (13.5; 19.98)	84.78 (81.02; 87.90)		22.59 (18.64; 27.11)
Higher education	8.30 (4.14; 15.97)	91.91 (82.58; 96.46)		19.35 (10.28; 25.94)
Household wealth quintiles				
Very poor	22.22 (16.84; 28.72)	77.78 (71.28; 83.16)	0.197	15.30 (12.49; 19.38)
Poor	18.41 (14.73; 22.76)	81.59 (77.24; 85.27)		24.30 (19.92; 29.30)
Middle	19.24 (11.89; 29.59)	80.76 (70.41; 88.11)		22.66 (18.30; 27.69)
Wealthy	17.61 (12.37; 24.46)	82.39 (75.54; 87.63)		19.49 (15.63; 24.03)
Very wealthy	9.48 (4.31; 19.59)	90.52 (80.41; 95.69)		17.93 (9.65; 30.88)
Sex				
Boys	22.75 (19.86; 25.93)	77.25 (77.25; 77.25)	0.005	43.59 (37.18; 50.21)
Girls	13.33 (9.20; 18.93)	86.67 (81.07; 90.80)		56.41 (49.79; 62.82)
Indigenous origin	17.50 (13.71; 22.07)	82.50 (77.93; 86.29)		
No	17.23 (13.70; 21.44)	82.77 (78.56; 86.30)	0.928	76.73 (71.93; 80.93)
Yes	17.50 (13.71; 22.07)	82.50 (77.93; 86.29)		23.27 (19.07; 28.07)
Area				
Urban	16.92 (13.39; 21.15)	83.08 (78.85; 86.61)	0.321	81.82 (75.86; 86.57)
Rural	19.76 (15.99; 24.18)	80.24 (75.82; 84.01)		18.18 (13.43; 24.14)
Domestic violence				
No	14.64 (11.27; 18.82)	85.36 (81.18; 88.73)	0.206	32.69 (27.59; 38.24)
Yes	18.79 (14.37; 24.19)	81.21 (75.81; 85.63)		67.31 (61.76; 72.41)
Maternal age (years) [mean (SD)]	28.27 (7.28)	29.93 (6.40)	0.011	29.64 (6.59)
Children age (months) [mean (SD)]	45.71 (7.85)	49.35 (6.75)	< 0.001	48.71 (7.08)

95%CI: 95% confidence interval; SD: standard deviation.

Table 1 shows the characteristics of the children with adequate and inadequate early childhood development. A total of 17.78% of children presented inadequate early childhood development, with boys showing a higher percentage of inadequate early childhood development than girls (22.75% and 13.33%, respectively). Overall, children and mothers in the inadequate early childhood development group tended to be younger than in the adequate group. A higher proportion of children with functional difficulties and disabilities had inadequate early childhood development than those without these problems (40.22% and 17.14%, respectively). The proportion of children with inadequate early childhood development was significantly higher for mothers with primary education or less (21.72%) and lower for those with complete tertiary education (8.3%) than for those with complete secondary education (21.23% and 16.49%, respectively). Children who lived in the poorest households (22.22%) and in rural areas (19.76%) had a higher prevalence of inadequate early childhood development than those from the wealthiest households (9.48%) and urban areas (16.92%). Finally, a higher prevalence of inadequate early childhood development was observed in children who were exposed to domestic violence than in those who did not (18.79% vs. 14.64%).

Table 2 shows the distribution of the environmental characteristics of the municipalities. Children with inadequate early childhood development lived in municipalities with a greater prevalence of alcohol consumption and a higher proportion of daily tobacco smokers than children with adequate early childhood development (alcohol: 51.96% vs. 50.53%; and daily smokers: 7.84% vs. 7.4%, respectively). Children with inadequate early childhood development lived in municipalities with lower population density than those with adequate early childhood development (an average of 1,908.12 vs. 2,789.69 inhabitants per km^2^). Other variables, although not statistically significant, were associated with early childhood development, including the marginalization index and the number of homicides.

**Table 2:** Characteristics for the municipalities of children with inadequate and adequate early childhood development.

	Early childhood development [mean (SD)]		p-value	Total [mean (SD)]
	Inadequate	Adequate		
Alcohol consumption (%)	51.96 (10.35)	50.53 (8.89)	0.158	50.77 (9.16)
Daily tobacco smokers (%)	7.84 (7.04)	7.40 (6.07)	0.454	7.48 (6.24)
Marginalization index	-1.19 (0.69)	-1.22 (0.68)	0.582	-1.22 (0.68)
Number of homicides	122.4 (190.44)	105.17 (148.28)	0.165	108.17 (155.79)
Population density by municipality (inhabitants per km^2^)	1,908.12 (3,847.94)	2,789.69 (4,444.87)	0.007	2,635.98 (4,389.02)

SD: standard deviation.

Table 3 shows the results of the multilevel model to estimate the association between early childhood development and alcohol consumption and tobacco use, adjusted by child- and municipality-level control variables. In the Model 1, the increase in one unit of proportion of daily tobacco smokers was associated with a 15.08% increase in the odds of inadequate early childhood development (OR = 15.080; 95%CI: 2.040; 111.400). A one figure SD increase in homicides was associated with a 17.9% increase in the odds of inadequate early childhood development (OR = 1.179; 95%CI: 1.051; 1.312), whereas a one-unit increase in population density was associated with 5.3% lower odds of having an inadequate early childhood development (OR = 0.947; 95%CI: 0.906; 0.989). In the Model 2, the increase in one unit of proportion of alcohol consumption was associated with an increase in inadequate early childhood development (OR = 13.410; 95%CI: 2.986; 60.240). A one SD increase in homicides was associated with a 19.6% increase in the odds of inadequate early childhood development (OR = 1.196; 95%CI: 1.053; 1.358). Population density was positively associated with adequate early childhood development since a one-unit increase in population density was associated with 5.4% lower odds of having an inadequate early childhood development (OR = 0.946; 95%CI: 0.904; 0.991). Finally, in Model 3, the increase in one unit of proportion of alcohol consumption was associated with an increase in inadequate early childhood development (OR = 9.415; 95%CI: 1.856; 47.770). A one SD increase in homicides was associated with a 19.4% increase in the odds of inadequate early childhood development (OR = 1.194; 95%CI: 1.057; 1.350). Similar to the previous model, the population density was also positively associated with adequate early childhood development; a one-unit increase in population density was associated with 5.4% lower odds of having an inadequate early childhood development (OR = 0.939; 95%CI: 0.897; 0.984).

**Table 3:** Adjusted associations between overall inadequate early childhood development and alcohol and tobacco consumption.

Predictor	Model 1 *		Model 2 *		Model 3 *	
	OR	95%CI	OR	95%CI	OR	95%CI
Proportion of daily tobacco smokers	15.080 **	2.040; 111.400			5.527	0.476; 64.220
Proportion of alcohol consumption			13.410 **	2.986; 60.240	9.415 **	1.856; 47.770
Number of homicides ***	1.179 **	1.051; 1.323	1.196 **	1.053; 1.358	1.194 **	1.057; 1.350
Population density by municipality ^#^	0.947 **	0.906; 0.989	0.946 **	0.904; 0.991	0.939 **	0.897; 0.984
Functional difficulties						
No	1.000 (Reference)		1.000 (Reference)		1.000 (Reference)	
Yes	1.013 **	1.005; 1.020	1.012 **	1.004; 1.020	1.012 **	1.004; 1.020
Maternal education						
Elementary or less	1.000 (Reference)		1.000 (Reference)		1.000 (Reference)	
Middle school	1.002	0.648; 1.550	1.002	0.650; 1.546	0.995	0.645; 1.534
High school	0.628 **	0.397; 0.992	0.617 **	0.390; 0.975	0.620 **	0.392; 0.981
Higher education	0.443 **	0.223; 0.880	0.430 **	0.217; 0.854	0.434 **	0.219; 0.861
Household wealth quintiles						
Very poor	1.000 (Reference)		1.000 (Reference)		1.000 (Reference)	
Poor	0.647	0.412; 1.015	0.614 **	0.390; 0.967	0.612 **	0.390; 0.962
Middle	0.654	0.385; 1.112	0.613	0.364; 1.032	0.611	0.364; 1.026
Wealthy	0.810	0.432; 1.517	0.778	0.413; 1.465	0.766	0.408; 1.440
Very wealthy	0.696	0.359; 1.349	0.653	0.333; 1.279	0.641	0.327; 1.255
Maternal age	0.976	0.948; 1.004	0.976	0.948; 1.005	0.976	0.948; 1.005
Sex						
Male	1.000 (Reference)		1.000 (Reference)		1.000 (Reference)	
Female	0.547 **	0.384; 0.779	0.546 **	0.384; 0.777	0.546 **	0.384; 0.777
Children age	0.927 **	0.904; 0.951	0.928 **	0.905; 0.952	0.928 **	0.904; 0.952
Domestic violence						
No	1.000 (Reference)		1.000 (Reference)		1.000 (Reference)	
Yes	1.493 **	1.030; 2.165	1.473 **	1.017; 2.132	1.481 **	1.023; 2.145
Median OR						
Null model	2.082	1.598; 2.567	2.082	1.598; 2.567	2.082	1.598; 2.567
Adjusted model	1.829	1.488; 2.170	1.877	1.521; 2.233	1.820	1.482; 2.159

95%CI: 95% confidence interval; OR: odds ratio.

* All models were adjusted by the set of individual variables for each child: sex, age in months, functional difficulties, maternal age, maternal education, household wealth quintiles, and domestic violence;

** Significant value (p < 0.05);

*** Re-scaled using z-scores, so that a one-unit change represents a one standard deviation change in municipal services of municipalities;

^#^ Per 1,000 inhabitants.

In addition, in all three models, several child-level control variables were significant. Children of mothers with complete secondary or tertiary education showed lower odds of presenting inadequate early childhood development than those who mothers had primary education or less; children with functional difficulties and/or children exposed to domestic violence in their household had greater odds of presenting inadequate early childhood development; girls had a lower risk of inadequate early childhood development than boys; and a one-unit (one-month) increase in children’s age was associated with an 8% decrease in the odds of inadequate early childhood development. In Models 2 and 3, children living in poor households had lower odds of inadequate early childhood development than those in very poor households.

Table 3 also show the values of the median OR both from the null model and from the adjusted models for child- and municipality-level variables. In the null model, the median OR was equal to 2.082 (95%CI: 1.598; 2.567), i.e., in the median case, the residual heterogeneity between municipalities increased by 2.082 times. In the adjusted models, the median OR decreased (OR_Model 1_ = 1.829; 95%CI: 1.488; 2.170; OR_Model 2_ = 1.877; 95%CI: 1.521; 2.233; and OR_Model 3_ = 1.820; 95%CI: 1.482; 2.159). Overall, the models fit indices indicated that the model fits the data well (p < 0.001).

## Discussion

This study investigated the association of the prevalence of alcohol consumption or tobacco use in Mexican municipalities with early childhood development for children under five years. Our findings revealed greater risks of inadequate early childhood development in children living in areas with higher prevalence of use of these substances. We found that greater alcohol consumption or tobacco use in Mexican municipalities was associated with higher odds of inadequate early childhood development. These results are consistent with a growing body of evidence suggesting that these factors are essential to understand developmental delays in children. Children of alcoholic parents have shown behavioral problems ^27^ ^,^ ^28^, which has been associated with increased cognitive and behavioral impairment in two recent meta-analyses. Likewise, a recent study from Brazil reported that the concomitant use of alcohol and tobacco increased the risk of motor and cognitive delays in children ^29^.

We found that living in a municipality with a greater number of homicides was associated with greater odds of inadequate early childhood development. Some studies suggest that neighborhood violence is an important risk factor for child development ^30^ ^,^ ^31^. Finkelhor et al. ^32^ demonstrated that exposure to several types of violence could influence children’s physical, social-emotional and cognitive development. Another analysis from the United States suggests that tobacco shops and off-sale alcohol outlets were proximal to violent crime activity, controlling for poverty, renters, resident mobility, and ethnic/racial heterogeneity ^33^.

Our results also showed that living in a municipality with a high population density was associated with decreased odds of inadequate early childhood development. Several studies found that children are healthier in urban areas that in rural areas ^34^. More densely populated places are more likely to have easier access to relevant health and education services for child survival and development. Furthermore, people in urban areas tend to be richer and better educated ^35^ ^,^ ^36^ ^,^ ^37^. However, these results should be interpreted carefully, considering that population density is a proxy for access to services and economic opportunities.

Within the individual level, age and female sex were associated with inadequate early childhood development. Children evaluated in this study showed that, as they grew, they had better results in the development of their abilities and skills, which corresponds to the continuous process of maturation where the development of various cognitive, communication, motor, and social-emotional skills is progressively built ^5^. Several studies examined gender differences, with boys usually presenting lower scores in certain areas of development ^38^ ^,^ ^39^. Mother’s education and household socioeconomic status have shown important influence on child development. Several studies found that mothers with a higher level of education have more intellectual and emotional tools to care for their children ^40^ ^,^ ^41^.

We also found that Mexican children with functional difficulties are more likely to have inadequate early childhood development. Children with disabilities often face barriers that affect their ability to participate fully and effectively in society, such as poverty, stigma, discrimination, violence, and limited access to programs ^41^. Finally, children exposed to violent discipline had higher risk of inadequate early childhood development. Black et al. ^5^ found that prolonged exposure to violent discipline may generate toxic levels of stress that affect development in the first years of life.

We highlight that our study presents several limitations. The first limitation is inherent to the cross-sectional study design, which does not allow for establishing causal relationships. Secondly, the environmental factors were measured at the municipality level since our ability to geocode the participants in the surveys was restricted to that level. Municipalities are fairly large and heterogeneous areas. However, the response rates of the two surveys were high and so were the sample sizes, which reduces the possibility of misclassification bias. Thirdly, we lacked information on the alcohol and tobacco consumption of the children’s parents, and whether the children were exposed at home. Therefore, we had to estimate the prevalence of consumption for each municipality using ENCODAT data with small area estimation methodology since the survey is not representative at the municipality level, which is widely used by statistical agencies in various countries. Finally, social determinants cover a wide spectrum of attributes, including housing circumstances; interpersonal interactions among children and parents; sociodemographic factors; day-care and school learning; among others. Our analysis partially covers all these compounds due to lack of availability of some variables in our database. Despite these limitations, our findings are consistent with prior research and this study served to identify potential associations between inadequate early childhood development and alcohol consumption and tobacco use, as well as other contextual factors. Future work based on longitudinal studies should try to confirm these associations with inadequate early childhood development.

## Conclusion

We found that children who lived in municipalities with higher exposure to alcohol consumption and tobacco use were more likely to have inadequate early childhood development in Mexico. Considering that some characteristics at municipality-level are notably modifiable by public health policies, all efforts should be taken to develop policies aimed at reducing alcohol consumption and tobacco use in municipalities to ensure adequate early childhood development, as well as reducing the number of homicides and emphasizing the importance of targeting these issues with effective interventions within neighborhoods.
